# A cool climate perspective on grapevine breeding: climate change and sustainability are driving forces for changing varieties in a traditional market

**DOI:** 10.1007/s00122-022-04077-0

**Published:** 2022-04-07

**Authors:** Reinhard Töpfer, Oliver Trapp

**Affiliations:** Julius Kühn-Institut, Federal Research Centre for Cultivated Plants, Institute for Grapevine Breeding Geilweilerhof, Siebeldingen, Germany

## Abstract

A multitude of diverse breeding goals need to be combined in a new cultivar, which always forces to compromise. The biggest challenge grapevine breeders face is the extraordinarily complex trait of wine quality, which is the all-pervasive and most debated characteristic. Since the 1920s, Germany runs continuous grapevine breeding programmes. This continuity was the key to success and lead to various new cultivars on the market, so called PIWIs. Initially, introduced pests and diseases such as phylloxera, powdery and downy mildew were the driving forces for breeding. However, preconceptions about the wine quality of new resistant selections impeded the market introduction. These preconceptions are still echoing today and may be the reason in large parts of the viticultural community for: (1) ignoring substantial breeding progress, and (2) sticking to successful markets of well-known varietal wines or blends (e.g. Chardonnay, Cabernet Sauvignon, Riesling). New is the need to improve viticulture´s sustainability and to adapt to changing environmental conditions. Climate change with its extreme weather will impose the need for a change in cultivars in many wine growing regions. Therefore, a paradigm shift is knocking on the door: new varieties (PIWIs) versus traditional varieties for climate adapted and sustainable viticulture. However, it will be slow process and viticulture is politically well advised to pave the way to variety innovation. In contrast to the widely available PIWIs, competitive cultivars created by means of new breeding technologies (NBT, e.g. through CRISPR/Cas) are still decades from introduction to the market.

**Supplementary Information:**

The online version contains supplementary material available at 10.1007/s00122-022-04077-0.

## The origins of grapevine breeding

Grapevine (*Vitis vinifera*) is the economically most important perennial fruit crop grown on 7,34 mil. ha (84,83 mil. t fresh grapes; OIV 2019) for wine grapes, table grapes, dry fruits, juice and other products made thereof. The cultivated *Vitis vinifera* ssp. *vinifera* and its wild relative *Vitis vinifera* ssp. *sylvestris* form the autochtonous species in Europe and the Near East, the Eurasian gene pool, largely endemic to the Mediterranean basin (Töpfer et al. 2011; Magris et al. 2021). The cultivated compartment of *Vitis vinifera* is highly susceptible to different pests and diseases, of which some were introduced into Europe (Feechan et al. 2013). Grapevine is propagated vegetatively and usually grown on rootstocks, that are tolerant to phylloxera, an insect pest introduced into Europe in 1863–1868 that almost destroyed European viticulture in the late nineteenth century (Galet 1977). Other serious pathogens were introduced from North America such as powdery mildew (PM, caused by *Erysiphe necator*) in 1845–1852 resulting in serious quality deficits, and downy mildew (DM, caused by *Plasmopara viticola*) in 1878 resulting in high yield losses (Galet 1977). Of minor importance in comparison to the mildews was the introduction of black rot (BR, *Guignardia bidwellii*) in 1885, which affects the grape yield (Galet 1977). All these pathogens dramatically changed viticulture and triggered the start of grapevine breeding activities in several countries.

**Fig. 1:**
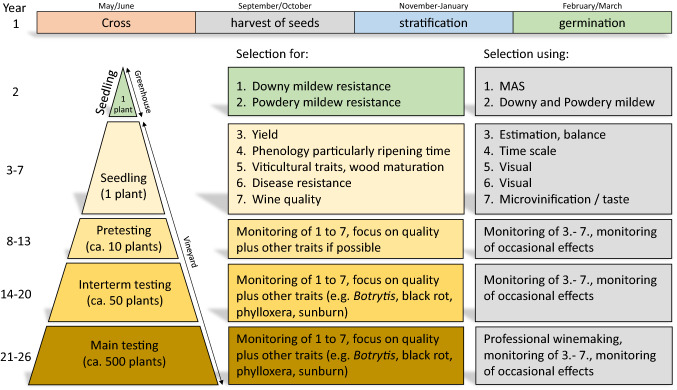
Steps of the grapevine breeding process at the JKI Geilweilerhof, their duration, the available number of plants and the respective selection criteria. Grapevine plants first yield grapes usually in year 3 of each testing step. Tests for wine quality are done for 3–4 years. Selection for wine quality is based on sensory evaluations. Due to the stepwise propagation of plants the possibilities to save time in the breeding process is limited and might in future be done by fusing pretesting and interterm testing. However, it is important, that final testings together with wine growers are done with very well pre-evaluated genotypes to avoid frustration in the wine sector

From a breeding point of view, the grapevine turned out to be a recalcitrant crop with its juvenile phase of about 3 years until the first fruit set, a long breeding cycle of about 25 years (see Fig. 1), high heterozygosity and strong inbreeding depression, the requirement for biotic and abiotic resistances, the general viticultural properties, and finally its complex product quality traits, especially wine quality. Advantageous for breeding is the interfertility of *Vitis vinifera* with other species of the *Vitis* gene pool: around 30 American species and about 40 Asian species (de Lattin 1957). Also advantageous is the huge gene pool of cultivars in ex situ collections (e.g. www.eu-vitis.de) and its rather small genome size of around 475 MB (Lodhi and Reisch 1995; Jaillon et al. 2007). Due to their vegetative propagation cultivars can be very old and few are in cultivation since centuries. Recently, Savagnin Blanc (VIVC 17636, Syn. Traminer) was found in a seed sample of an archaeological excavation in southern France dating back around 900 years (Ramos-Madrigal 2019). Other cultivars like Pinot Noir, Heunisch (first mentioning 1283), or Riesling (first mentioning 1435) are centuries old, too. Over time, cultivars were selected for their particular, well received tastes and beneficial viticultural properties. Today, ten cultivars account for 26% of the vineyards worldwide (Magris et al. 2021).

All the traditional wine grape cultivars of the species *Vitis vinifera* are lacking resistances against PM and DM. Both diseases are now widespread in all wine-growing regions around the world and are controlled by intense plant protection regimes (Phytowelt 2003). As consequence of the huge damage caused by the introduction of the pathogens, many breeding activities were initiated in Europe at the end of the 19th and beginning of the 20th century. In a first step, European breeders used North American *Vitis* species or interspecific hybrids of North American species with *Vitis vinifera*, known as American Hybrids. Decades later, after initial setbacks, offspring of crosses between North American *Vitis* species resulted in tolerant rootstock cultivars that solved the phylloxera crisis. The combination of scions of *V. vinifera* cultivars, which are generally resistant to phylloxera apart from their roots, grafted onto a phylloxera tolerant rootstock results in a grafted vine, that is sufficiently robust to survive phylloxera feeding. Until today, this concept keeps viticulture as we know it alive.

## New varieties faced disapproval

In order to ensure safe and sustainable viticulture and to reduce the high need for fungicides, breeders began to introduce resistances against the mildews from North American and later from Asian *Vitis* species into the genepool of *Vitis vinifera*. The introgression proved to be difficult, due to inbreeding depression, long generation time, and the complexity of the desired traits, in particular a deficiency in wine quality inherited from the wild species (Hedrick and Anthony 1915). In contrast to the phylloxera problem, the genetic contribution to solving the mildew problems by breeding was delayed. Two main problems arose: (1) the early discovery of the fungicidal effects of sulphur against PM and copper against DM reduced the pressure on viticulture (Millardet 1885) and (2) more relevant for viticulture, wine quality deficits in the early breeding lines and first resistant varieties (the so-called French hybrids), which were developed in the late 19th and early 20th century, resulted in a lack of acceptance and political ban. Ribéreau-Gayon (1960) found the anthocyanin malvidin-3,5-diglycoside predominantly in wines made from French hybrids and used it as an easily detectable marker for blends with hybrid wines of lower quality. His 2D-paper chromatography technique was used to proof blending with the consequence of a rapid decline of growing area in France of hybrid varieties from a peak of about 402.000 ha (Galet 1988). Moreover, in subsequent decades the poor wine quality image of American and French Hybrids was transferred onto all grapevine plants resulting from resistance breeding and became a general prejudice against resistant varieties. Despite of good resistance properties and well tasting grapes in many French hybrids, their wine quality deficits become evident after fermentation. The fermentation and later on chemical reactions in the wine change the matrix of a sweet juice converting it into an alcoholic wine with totally altered sensory attributes. In a juice, sweetness and primary fruit aromas often cover metabolites or resultant derivatives, which arise upon fermentation. Many aroma compounds in the berry are present in a bound (e.g. glycosylated), odour-imperceptible form which are later responsible for the variety bouquet. However, some off-flavours are well recognizable at the juice level as “hybrid” or “foxy” taste e.g. due to furaneol (4-Hydroxy-2,5-dimethyl-3-furanone), methyl anthranilate, 2-aminoacetophenon (Rapp et al. 1992; Rapp and Versini 1996), others are recognizable in nanogram concentrations like mercaptans or thiol-based compounds. The weather or stress conditions can strongly influence aroma formation in grapes resulting in off-flavours in years of particular growing conditions (e.g. in cv. Pollux; Rapp et al. 1992). This sensitivity of the wine growing sector concerning quality issues in new grapevine cultivars (Teissedre 2018), made breeders particularly attentive and made them rigorously select breeding lines without off-flavours. Thus, the general prejudice of poor taste in selections from resistance breeding is a matter of the past. For these new cultivars, the term “hybrids” is misleading and should be avoided at least in a scientific context.

Alexis Millardet suggested 1880 to combine quality of the European wine grape with the resistance of American wild species. It took around 120 years until the first convincing cultivars coming from grapevine breeding programmes emerged on the market (compare Table 1). After having achieved this breakthrough further cultivars appeared with some of them carrying resistances from the Asian gene pool. It was the continuous effort of many breeders, that led to a success over generations. Today, breeding programmes for wine grapes are running e.g. in Germany at WBI Freiburg i.Br, JKI Geilweilerhof, HGU Geisenheim, and LVWO Weinsberg (Ruehl et al. 2015; Weinmann et al. 2019), in France at the INRAE Colmar (Schneider et al. 2019), in Italy at University Udine, at the Fondazione Edmund Mach, at University of Milan (Bavaresco et al. 2015), in Switzerland at Agroscope Changins (Spring and Dupraz 2021) and by the private breeder Valentin Blattner (Blattner 2006). A comprehensive overview including programmes in other continents is given in the book of Andrew Reynolds (2015) on Grapevine Breeding Programs for the Wine Industry and other publications (Buonassisi et al. 2017; Dry et al. 2019). The strength of the different breeding programmes is that they are located in different environments and thus provide advantages to select for different traits. All these efforts resulted in a number of cultivars, which can be attributed to the first generation of new varieties with high wine quality (Teissedre 2018) and resistance properties paving the way for improving the sustainability in viticulture within the next decades.

**Table 1:**
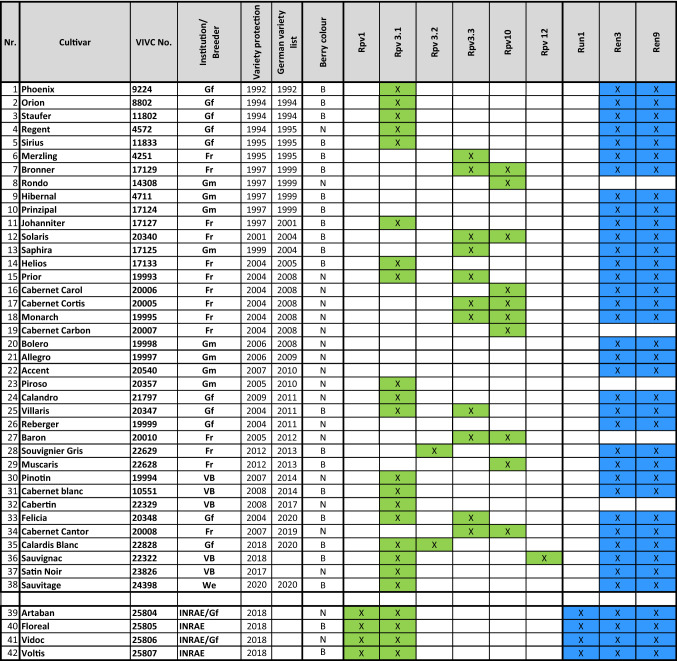
A collection of 42 resistant cultivars available in Germany and France including the presence of different resistance loci against downy mildew (Rpv) and powdery mildew (Run and Ren) (colour table online)

Ren1 and Ren4 were also checked but are absent from all tested cultivars. “Variety protection” lists the year of protection in Germany or the EU, whereas “German variety list” states the year of introduction to the national German variety list. Berry colour is stated as white (B) or black (N). Gf = JKI Geilweilerhof; Fr = WBI Freiburg i.Br; Gm = HGU Geisenheim; VB = Valentin Blattner/Rebschule Freytag; We = LVWO Weinsberg; INRAE = INRAE Colmar. Further information on the used markers in Table S1

## Future needs: new resistant cultivars for viticulture

Despite a lot of disappointments during the long history of grapevine breeding, its success is visible today, new cultivars are constantly entering the market and the demand for new cultivars is increasing. A number of factors force viticulture to open itself for variety innovation:

- The need to increase sustainability:
  - Loss of active fungicidal compounds: fungicide resistant pathogen strains (Alzohairy et al. 2021; Campbell et al. 2021) and loss of official authorizations for different fungicides due to stricter regulations (Harzer 2021)
  - Political debate on the European Green Deal 2050 and its Farm to Fork Strategy claiming to reduce the overall use and risk of chemical pesticides by 50% until 2030 (European Commission 2020) and simultaneously raising public demand for more sustainable food production (e.g. by the Fridays for future initiative)
- Climate change and its consequences for viticulture (e.g. Ausseil et al. 2021; Bernath et al. 2021; Biss and Ellis 2021):
  - In particular white wine cultivars lose the typicality of their wine style but red wine cultivars could be the winners in cool climate regions
  - Extreme weather events and effects on biotic and abiotic stress.

Today, viticulture without protecting grapevines against the mildews is not possible (Warneke et al. 2020; Campbell et al 2021). Sulphur, copper, and synthetic fungicides are the tools to manage diseases. Plant protection in viticulture requires good concepts and should be limited to the essential level as it is well known that intense synthetic fungicide applications in agriculture select for pathogenic strains, which acquired a resistance against active compounds (Campbell et al. 2021). Therefore, in viticultural practice, plant protection regimes should change the active compounds with each fungicide treatment to reduce the chances of formation of fungicide resistances. This biological challenge is facing another rather unexpected challenge: the loss of approval of certain active compounds of fungicides. This loss of pesticides within the plant protection portfolio becomes even more challenging as it conflicts the resistance management concepts (Lykogianni et al. 2021).

Due to climate change weather extremes increase in frequency. In Germany, for example, there have been extreme weather events throughout the growing season in recent years affecting viticulture:

- 2016 was very rainy and wet resulting in DM epidemics and yield loss
- 2017 early bud burst was followed by a late spring frost with strong impact on yield
- 2018 hot and dry with alcohol rich white wines and untypical aromas; sunburn on some varieties
- 2019 hot and dry with alcohol rich white wines and untypical aromas; sunburn on some varieties
- 2020 hot and dry with alcohol rich white wines and untypical aromas; sunburn on some varieties
- 2021 was very rainy and wet resulting in the up to now strongest DM epidemics and yield loss

Located on the northern border of viticulture, southern European varieties, which during the last decades usually did not fully ripen in Germany, behave increasingly better. They become an alternative for wine growers hoping that their marketing is easier compared to new cultivars. However, in growing these varieties sustainability is not improved as they do not carry mildew resistances.

## Resistant cultivars need plant protection

It is clear that the story is not that simple and not black and white. Current resistant cultivars (examples in Table 1) permit a reduction in fungicides between 50 and 80% on average and depending on the environmental conditions. They are the long anticipated contribution to sustainability, which fulfils the EU Green Deal requirements. However, resistance in plants does not mean immunity. A major issue in resistance breeding is the question of durability of resistance. In order to increase the stability and durability of resistance, there are different strategies of resistance deployment in agriculture (Rimbaud et al. 2018): crop rotation, mixtures (different cultivars in the same field), mosaics (different cultivars in adjacent fields) and the stacking of resistances in the same cultivar. As grapevine is a perennial crop and viticulture has a long turnover time of 30 years on average for a field, the most applicable strategy for viticulture is increasing the stability and durability of resistances by stacking resistance loci (also known as “pyramiding resistances”). The need for such a consideration is shown by Peressotti et al. (2010) which reported the breakdown of the resistance Rpv3.1 in cv. Bianca making a more general consideration for future viticulture necessary. Plenty examples can be given of breaking gene-for-gene resistances in cereals (Brown 2015 and literature cited therein). In the United Kingdom considerable progress for durable resistance in spring barley breeding was achieved by selecting loss of function *mlo* alleles considerably reducing the severeness of powdery mildew (*Blumeria graminis* f. sp. *hordei*). In contrast, in winter wheat the resistance against *B. graminis* f.sp. *tritici* is partial and apparently of polygenic nature consisting of several minor alleles (Brown 2015).

The breeding cycle in cereal breeding is much faster than in grapevine breeding and the genetic understanding of resistance in cereals is much more advanced compared to grapevine. As a consequence, as long as new options remain to be developed, increasing the stability and durability of resistances in grape will be addressed by pyramiding resistance loci (see Table 1). However, genetic contributions and management practices need to be adapted. Breeders communicate these concepts for the cultivation of resistant cultivars and an adapted chemical plant protection needs to be elaborated. Recently, a first report of a downy mildew strain immune to stacked resistances Rpv3.1 and Rpv12 emphasize the need for additional plant protection in order to maintain resistance properties of new cultivars (Wingerter et al. 2021). A “no plant protection” agenda is not the solution indicated by these findings and other reasons. However, plant protection should and can be reduced to the minimum necessary in order to safeguard the environment by using resistant cultivars. Despite of a residual plant protection requirement, it is a significant contribution if instead of 10 or more treatments, 2 to 3 treatments (between flowering and bunch closure) are sufficient. Most important: such new cultivars are ready for the market and can be grown today!

The reduction in fungicide applications against the mildews resulted in the emergence of other “minor” pathogens such as *Guignardia bidwellii* (causing black rot) (see below). Such secondary effects could result in emerging diseases, which can be controlled by a minimum of treatments. This indicates that grapevine breeding can only provide one answer towards the challenges ahead of viticulture.

## New mildew resistant cultivars for the 21st century

In Germany more than 38 new cultivars are available for winegrowers today (Table 1) and more are comming. Several of these are also available for winemaking in France, together with four new so far exclusive cultivars. Pedigree information on these cultivars has been elaborated and verified by (Röckel and Maul, data not shown) and can be found in the *Vitis* International Variety Catalogue, *V*IVC (Maul et al. 2021). Due to the sensitivity of the viticulture sector on wine quality issues, it is important to state, that all of these cultivars are essentially free of off-flavours. Doubtless, many of these cultivars proved to have wine quality characteristics, which in blind tastings of trained panels make them indistinguishable from the wine quality of traditional cultivars. This clearly indicates the substantial breeding progress achieved in recent decades. In terms of resistances, however, there is space for improvement. All 38 “German” cultivars listed in Table 1 were selected without using marker assisted selection (MAS), i.e. purely based on phenotypic evaluations. A marker analysis was performed to show which resistances have been inherited. The results are presented in Table 1 (for markers and sequences used, see Table S1). The two French/German and two French cultivars are the first, which were initially preselected by MAS for resistances. As a control, two resistance loci, Ren1 and Ren4, were analysed in all the cultivars but they are not present in this set of cultivars as expected (Trapp and Eibach, data not shown). The table indicates the tested loci but further unknown loci might exist in these cultivars.

**Fig. 2:**
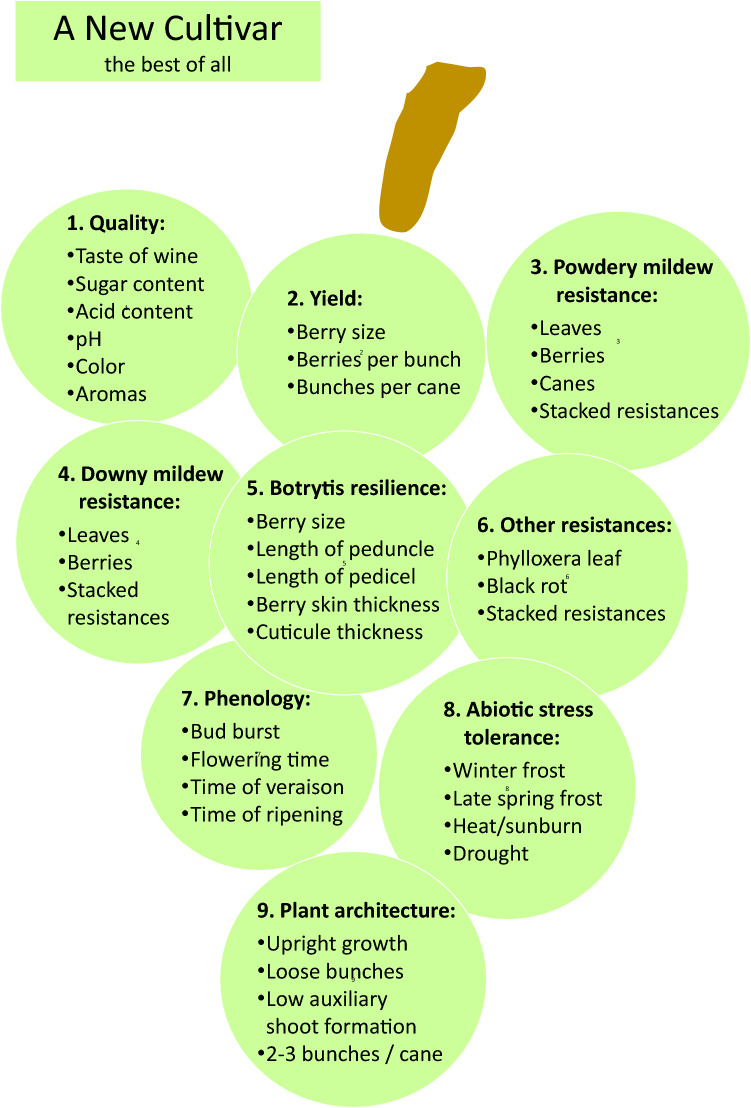
Most relevant traits to be accumulated in new grapevine cultivars. Importance of traits is indicated by numbering. Of particular complexity is the trait “quality” which is understood as quality of the wine. A new cultivar should have the best of all traits

**Fig. 3:**
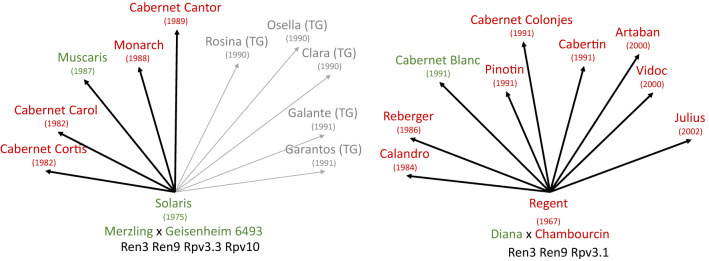
Solaris and Regent represent founder varieties for a number of further cultivars. Arrows indicate the parent–child relations; “grey” represents the relation to table grape (TG, grey) cultivars; “Green” depicts white wine cultivars; “red” is for red wine cultivars. VIVC numbers are given in Table S2 (colour figure online)

Ren3/Ren9 (Zendler et al. 2017) are the common loci to the cultivars of all breeding programmes carrying PM resistance as indicated in Table 1. First cultivars that carry an additional PM resistance introgressed from *Muscadinia rotudifolia* are from INRAE/Gf and INRAE. This locus confers a double resistance against PM (Run1) and DM (Rpv1) (Feechan et al. 2013). DM resistances in Table 1 are conferred by loci of various origin. Most widely distributed is Rpv3.1 but also related alleles are found in the cultivars (Di Gaspero et al. 2012). Rpv3 loci are today regarded as resistance loci with medium strength. A combination of different Rpv3 loci, however, shows stronger resistance effects than the single loci. The breeding programmes at Freiburg i. Br. and Geisenheim also used the resistance Rpv10 (Schwander et al. 2012) originating from the Asian species *V. amurensis*, which proved to be a rather strong locus. The locus Rpv12 was similarly introgressed from *V. amurensis* and turned out to be a strong locus as well (Venuti et al. 2013; Müllner et al. 2020). There are more resistance loci known for PM and DM. Hausmann and Töpfer (2021) provide an occasional updated overview on loci identified in the *Vitis*/*Muscadinia* genepool (www.vivc.de/loci). It is noteworthy that from 13 PM loci (Ren/Run) known only three (Ren3/Ren9 and Run1) are found in wine grape cultivars up to now (Table 1) indicating the delay between research and breeding application. For DM 31 loci are described and four loci are used in today´s cultivars (Rpv3 (different alleles), Rpv10, Rpv12, and Rpv1) (Table 1). It is important to mention, that the cultivars in Table 1 were thoroughly selected by their respective breeders, meaning they show the combination of quality and resistance together with most of the other relevant viticultural traits (see Fig. 2) and form an excellent starting point for further breeding efforts. Breeders have realized this option and created a number of new cultivars based on first resistant cultivars. Figure 3 shows Solaris and Regent as two main founder cultivars. That means on the other hand, that the gene pool is already rather narrow and new genetically independent cultivars are desired. In a first step, the introgression of new resistances into the genepool is required in order to combine and accumulate favourable alleles for desired traits (e.g. yield, ripening, quality parameter) prior to be ready for the selection of a new cultivar.

## New resistance loci but also new challenges

To come to genetically independent new cultivars an exchange between different breeding programmes is necessary. Furthermore, other loci have to be recruited from the pool of identified resistances and the search for new loci must continue. Further strong loci for PM resistance are expected to be introgressed soon into the gene pool of *V. vinifera* like Ren1 (Hoffmann et al. 2008) from the table grape gene pool and Ren4 from *V. romanetii* (Ramming et al. 2011). Additionally, researchers all around the globe work hard on finding new resistance loci in the *Vitis* genus, which could then be used for future introgressions into *Vitis vinifera*. However, due to phytosanitary reasons, the global exchange of genetic resources of *Vitis* is a complex, expensive and time consuming task, impeding fast adoption of new resistances into breeding programmes worldwide, unless the vine is already in the breeder’s hands. The stepwise introgression of new resistances by performing several pseudo backcrosses with *V. vinifera* cultivars or elite breeding material permits the elimination of undesired characters particularly quality deficits from wild relatives. An outstanding example was the introgression of the Run1/Rpv1 locus by Alain Bouquet (Pauquet et al. 2001) from *Muscadina rotundifolia* up to pBC4 and pBC5. Such introgression lines were used to make crosses to combine the Run1/Rpv1 locus with other resistance loci (Ren3, Ren9, Rpv3.1) found in cultivars Regent and Villaris. The resulting offspring produced the cultivars Artaban, Vidoc, Floreal, and Voltis (Schneider et al. 2019). The stacked resistances in these cultivars are the start for a new generation of resistant cultivars. Some of the current cultivars like Calardis Blanc show further positive traits like black rot resistance, botrytis resilience or tolerance to sunburn. Black rot became of major relevance in recent years (Lipps and Harms 2004; Molitor and Beyer 2014). In some locations at the Mosel valley in 2002, plants that carry resistances against PM and DM became infected by *Guignardia bidwellii* upon reduction of fungicidal treatments. Quickly the demand for resistant plants was claimed. Figure 2 shows the main breeding goals in wine grape breeding according to their priority. In addition, new challenges are arising and might be considered in future cultivars: e.g. minor or new pests and diseases like phomopsis (Barba et al. 2018), anthracnose (Li et al. 2021), or *Xylella fastidiosa* (Pierce’s disease) (Riaz et al. 2011; Cendoya et al. 2020). As climate change creates a major impact on viticulture, flowering and ripening of grapevine occurs earlier in the season. Plants are endangered from late spring frost, from drought stress and sunburn, and from conditions that change drastically the style of the wine, which tend to become rich in alcohol and untypical in their flavour expression. As consequence, breeders redefined their breeding goals to give answers to these challenges and to select for robust and climate change adapted plants. However, to manage all these wishes and find a good compromise becomes more and more challenging and new strategies need to be developed to make grapevine breeding more efficient.

## Limits and options for the acceleration of breeding

Increasing the number of seedlings in order to increase the chances to find the desired combination of traits in a plant leads to logistic limitations: handling of seedlings in the greenhouse and vineyard is limited by space and labour force in breeding programmes. Furthermore, creating F1-seeds by making more crosses in the short period of flowering is also a limitation. Assuming more F1-seeds can be created, the most straight forward strategy for selecting seedlings is the application of MAS to identify desired combinations of resistance loci. Several resistance loci can be selected by MAS but still, the pool of loci available for MAS is limited at present as many resistance QTLs are published but breeders often lack tightly linked and easily usable markers for MAS. As a consequence new and easily usable markers for additional resistance loci and for other traits (yield, phenology, abiotic stress, quality parameter etc.) need to be developed. We need to recognize, that it remains a huge task to develop markers, that are easily applicable in MAS in order to increasing breeding efficiency of the breeding programmes. Additionally, genomic selection (GS) as a tool to raise efficiency in breeding programmes for many crops species, is still in its infancy in grapevine, although its usability has been demonstrated in table grapes (Viana et al. 2016). To create new varieties with high wine quality, already Hedrick and Anthony (1915) stressed the point, that the better the parent´s quality the higher the chances to obtain good quality offspring genotypes. Therefore, it is important to introgress a novel resistance locus into a high quality *V. vinifera* background by consecutive pseudobackcrosses in order to start with elite lines in crosses for new cultivars. This introgression can be assisted by molecular markers (marker assisted backcrossing, MABC) to select for a rapid reduction of genomic regions of the resistance donor but finally need further evaluation. With an increasing number of loci stacked in future varieties, the introgression should be as small as possible to avoid a linkage drag. In addition, the use of microvines might be helpful (Pellegrino et al. 2019) for introgression but selection for other traits remains difficult due to the dwarf phenotype.

## Locus specific homozygous (LSH) lines—a new concept

**Fig. 4:**
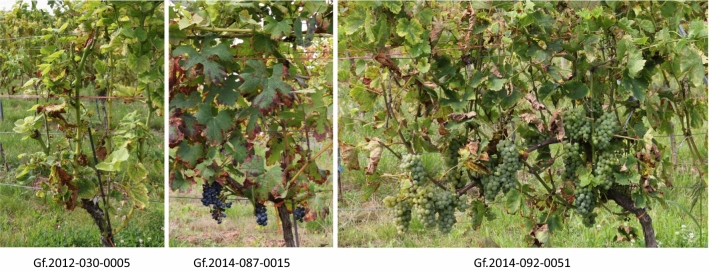
LSH-Line Gf.2012–030-0005 suffering from inbreeding depression and no fruit. LSH-line Gf.2014-087–0015 shows a deficit in yield. The well performing LSH-line Gf.2014–092-0051 shows sufficient vigor and yield, the juice and fermented wine is essentially free of off-flavours. Pedigrees are given in Fig. 5. High resolution images can be found in the supplemental Figures S1-S3

**Fig. 5:**
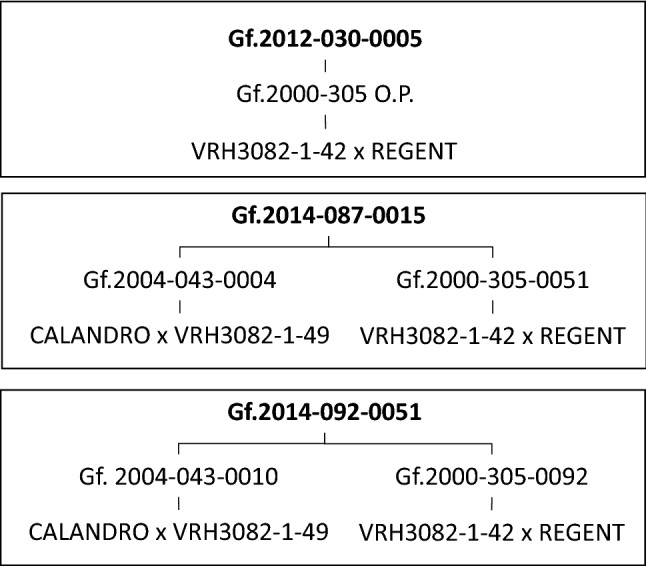
Pedigrees of (locus specific homozygous) LSH lines Gf.2012–030-0005, Gf.2014–087-0015, and Gf.2014–092-0051 which homozygously carry the loci Run1/Rpv1, Ren3, Ren9 and Rpv3.1

A novel approach to get stacked resistances could be the use of locus specific homozygous (LSH) breeding lines as proposed by Töpfer and Eibach (2017). These lines are homozygous only for selected resistance regions and thus the entire offspring will be resistant. A selection for the resistance loci in the offspring by MAS is not necessary any more. In a first attempt, breeding lines carrying Run1/Rpv1, Ren3, Ren9, Rpv3.1 were selfed and the offspring was analysed for genotypes homozygous for the resistance loci (Fig. 4, for pedigrees see supplement 2). The high inbreeding depression known for grapevine became a problem with this approach. Most of the genotypes did not proof to be vigorous enough or were not fertile. LSH line Gf.2012–030-0005 (Fig. 4) is such an example. It shows stunted internodes and does not produce grapes. Therefore, crosses of breeding lines showing good characteristics and different genealogy but identical resistance loci were chosen as parents (Fig. 5).

The offspring turned out to be more vigorous and inbreeding depression was overall a minor problem. However, a variation exists in these seedlings as shown in Fig. 4 as Gf.2014–087-0015 shows a deficit in yield while Gf.2014–092-0051 proved to be vigorous and good yielding. Very important is the observation that neither juice not the fermented wine carry any off-flavours. In wine tastings the genotype Gf.2014–092-0051 in fact shows a wine of overall good characteristics. Such good performing LSH-lines could be a very good contribution to develop new cultivars with stacked resistances. The entire F1-offspring of such crosses can be selected for all other traits as it is uniform for the resistance loci of the homozygous parent. For example a cross of LSH Gf.2014–092-0051 (Fig. 4) with Calardis Blanc [Ren3, Ren9, Rpv3.1, Rpv3.2 and a resistance against black rot (Rgb, the locus needs to be characterized)] would result in an offspring that carries in all F1-plants Run1, Ren3, Ren9 for PM and Rpv1, Rpv3.1 and in 50% of the F1-plants the Rgb locus against black rot. Of the F1-plants 25% carry the Rgb locus and would have inherited the Rpv3.2 of Calardis Blanc, too. Homozygousity for Ren3, Ren9 and for Rpv3.1 will occur in the F1-generation of such a cross as well. Moreover, good performing LSH-lines with high wine quality are very suitable partners for crosses with traditional *Vitis vinifera* cultivars. The idea behind such crosses is the combination of two high wine quality genotypes without any further consideration of resistances in the offspring. Moreover, if such LSH-lines are selected for female flowers, crosses and thus the production of F1-seeds becomes easier as no emasculation of the female parent is necessary. The development of LSH-lines is time consuming. It could be done in parallel to variety selection as a branch of a breeding programme. In order to minimize a genetic bottleneck, genetic diversity of LSH-lines becomes very important.

Genetic improvement by new breeding technologies (NBT).

Another important option to improve grapevine breeding are the new breeding technologies, first and foremost the CRISPR/Cas system, which allows the introduction of genomic changes in existing cultivars at sites of interest while the rest of the genome remains unchanged (“genome editing”; Jinek et al. 2012). In this way a specific mutation or targeted change of specific properties of a cultivar can be achieved in a short time frame, even without the introduction of foreign DNA. This type of generation of variation, is different from the introduction of resistance genes e.g. via *Agrobacterium tumefaciens*, which aside of classical breeding was the main way of creating resistant crop plants for decades, though largely banned. The most common application of the CRISPR/Cas system is the knockout of genes relevant for traits of interest, but also more sophisticated approaches into genome editing exist for the application in agriculture (Zhu et al. 2020; Rönspies et al. 2021). CRISPR/Cas opens up new opportunities for grapevine breeding as specific mutations can be introduced, thus modifying known cultivars without changing other characteristics. This is of particular interest as in many cases the marketing of wines is strongly associated to the variety name. These genome edited plants resemble spontaneous variants of cultivars, well known from clonal selection. As long as register traits are not changed, the identity of the variety is not changed. Resistance traits are typical non-register traits. If register traits (like bunch density, beginning of ripening, shape of the leaf blade, and so on) are affected, the varietal identity might be lost. In this case, the plant—according to current legislation relying on the DUS criteria (Distinctness, Uniformity and Stability according to UPOV 2002)—is no longer treated as equivalent to the initial cultivar which was previously defined according to its register traits. This fact limits the range of variability of register traits in varieties, irrespective of whether they are of spontaneous origin or induced by NBT.

However, for NBT approaches the genetic basis for the traits in question needs to be clear and well understood. Today, reports of genome edited grapevines mostly focus on the introduction of resistance against powdery mildew and botrytis by knocking out known susceptibility genes (S-genes) or putative S-genes derived from published work in other organisms (Wang et al. 2018; Wan et al. 2020; Pirello et al. 2021). While studies were successful in genome editing and even enhancing resistance properties, there are no edited cultivars made with CRISPR/Cas or a similar technique available on the market right now or can be expected in the near future as there are some hurdles:

1. The present EU legislation defines genome edited plants as GMOs and customers are refusing such products. Therefore, genome edited cultivars are currently not ready to be accepted by the market.
2. The genetic background of the traits of interest is often unknown or poorly understood. In order to be able to change a wealth of properties in grapevines, substantial progress in understanding grapevine genetics is needed.
3. Currently, the number of cultivars accessible for biotechnological approaches is low and focussed in the past on Chardonnay (Malnoy et al. 2016; Ren et al. 2016), and table grape varieties like Thompson Seedless (Li et al. 2020; Wan et al. 2020; Wang et al. 2021). The addition of new cultivars into editable cell cultures, their editing and the regeneration of plants is usually difficult and very labour intense. Today, the spectrum of grapevine cultivars that can be edited is quite narrow.
4. Finally, a single genome edited plant with the desired trait needs to be propagated for several cycles in order to be able to provide the market with sufficient plant material (grafted vines). After the successful regeneration of edited plants, the setup of propagation fields is going to take at least 8–10 additional years, reducing the time saving potential of breeding with new breeding technologies, whereas in the classical cross breeding scheme, several propagation steps are already included.

Taken together, it is safe to say, that the possibilities of the CRISPR/Cas system are near endless and it is worth exploring its potential for grapevine breeding. Given our current knowledge of grapevine genetics and the gene trait relations and biotechnological possibilities it is however not foreseeable when edited cultivars will reach the market.

## Market introduction is an unsolved challenge

Regardless of all the breeding success and expected further resistant and improved new cultivars, acceptance needs to be achieved in the market. The rational arguments are clear:

- viticulture must become more environmental friendly
- viticulture is losing active fungicidal substances to protect the plant,

The truth is also that

- winemakers stick to traditional cultivars (all highly susceptible for mildews) as marketing mostly relies on varietal naming
- winemakers have a conflict in the terminology to advertise wines made from fungus-resistant vines
- preconceptions address the new varietal names.

On average about 25 years are required from a cross to a protected variety unless parts of the selection are transferred to the wine growers. If wine growers are disappointed by growing insufficiently tested genotypes as they experienced in the first half of the 20th century for the French Hybrids market entry is hindered. Regardless, it remains a challenge to bring new cultivars to the market. For German consumers, the most important criterion when buying wine is the “price,” followed by the “grape variety” as well as the “reduction in pesticides”, and “carbon emissions.” The attribute “better for the environment” has the least relevance (Nesselhauf et al. 2020). The study shows that for German wine consumers—and possibly for wine consumers in general—environmental aspects are not taken into account when making a purchase decision. When making a decision for the luxury good wine, the consumer does not want to deal with an unpleasant, conflictual topic. This raises the question of whether the topic of environmental protection, which has been the topic of breeders for decades, is suitable for communication. As a consequence, winemakers usually avoid the issue of crop protection when marketing their wines, which rationally should be the selling argument of new varieties.

Hence, it is tempting to speculate as to whether another way of communication is easier for both parties. The weather is the everyday issue in human life. Everyone is affected by weather extremes, extreme heat, drought, lots of rain, etc. This personal experience could be used to talk about new varieties adapted to climate change, a term which describes the difficulties viticulture faces all-encompassing and does not only focus—but includes—plant protection. Consumer interest in new varietal wines can thus begin in a conflict-free space and be an enriching discovery. At this point, it is a challenge for marketing experts to develop a suitable communication strategy. The varieties are available and more will come.

## Conclusion

From the seventeenth century on the European settlers in the new world tried to cultivate *Vitis vinifera* cultivars for more than 200 years but finally failed due to pathogens and environmental conditions. Attempts to hybridize North American wild species and *Vitis vinifera* were quite successful but these American hybrids had a particular taste known as hybrid taste. Further backcrossed with *Vitis vinifera* resulting in the French hybrids showed substantial breeding progress, which, however, was not sufficient to reach the quality level of traditional wine grape cultivars. Additional crosses were necessary to achieve an introgession of resistances into *Vitis vinifera* without negative impact on wine quality. By the end of the 20th century, a first generation of convincing cultivars entered the market. The next generation of cultivars improved for resistance is currently growing in the breeders vineyards. They need to fulfil further requirements driven by climate change, sustainability and reliability of the production process. A compromise at the quality level will neither be possible nor necessary. Genome editing in grapevine is today still in its infancy. It will take at least two more decades for the first edited plants to reach the status of current traditionally bred new cultivars in terms of market readiness. Resistances and climate change adaptation are the driving arguments to get NBTs tested and to extend the breeders tool box. They are expected to become an indispensable additional tool on the long term.

Given the long lifespan of a vineyard (30 years and more), renewing it is the best opportunity to switch to a new variety. In Germany, 25% of the area under cultivation will be replanted within 10 years. There is a chance to focus on sustainability. We have to stop with dogmatic pros and cons of traditional versus new varieties or categorically reject NBTs as a tool and take a first step. Agriculture is a key player for better environmental protection, and viticulture is a small but important sector in terms of contributing to sustainability.

## Supplementary Information

Below is the link to the electronic supplementary material.

Supplementary file1 (PDF 1650 kb)

Supplementary file2 (XLSX 11 kb)

Supplementary file3 (XLSX 9 kb)
