# Supplementary figures and images for: A cool climate perspective on grapevine breeding: climate change and sustainability are driving forces for changing varieties in a traditional market
Source: Theor Appl Genet. 2022 Apr 7;135(11):3947–60. doi: 10.1007/s00122-022-04077-0 (PMC9729149; doi:10.1007/s00122-022-04077-0)

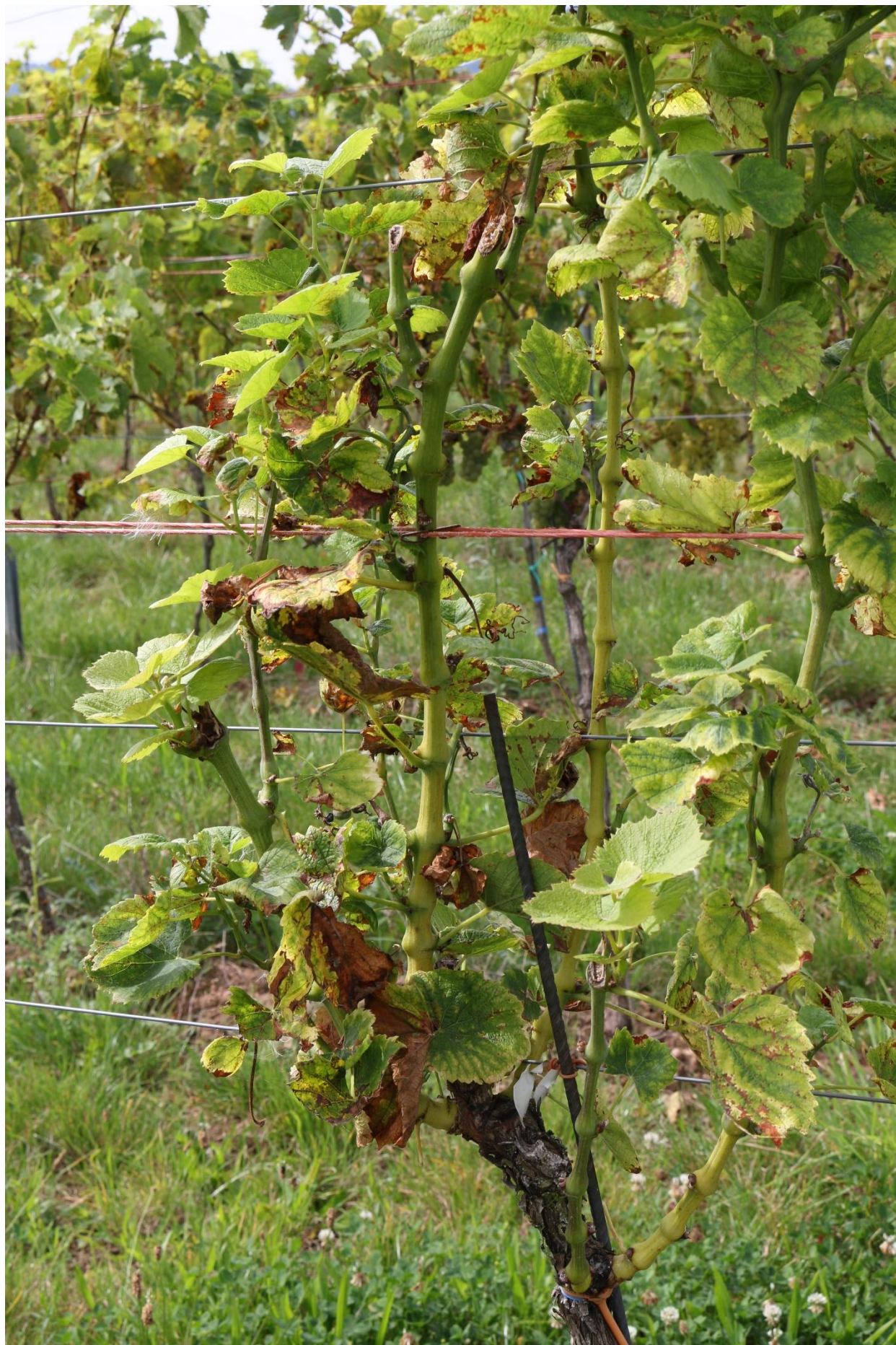

Figure S1: Vine of the LSH line Gf. 2012-030-0005

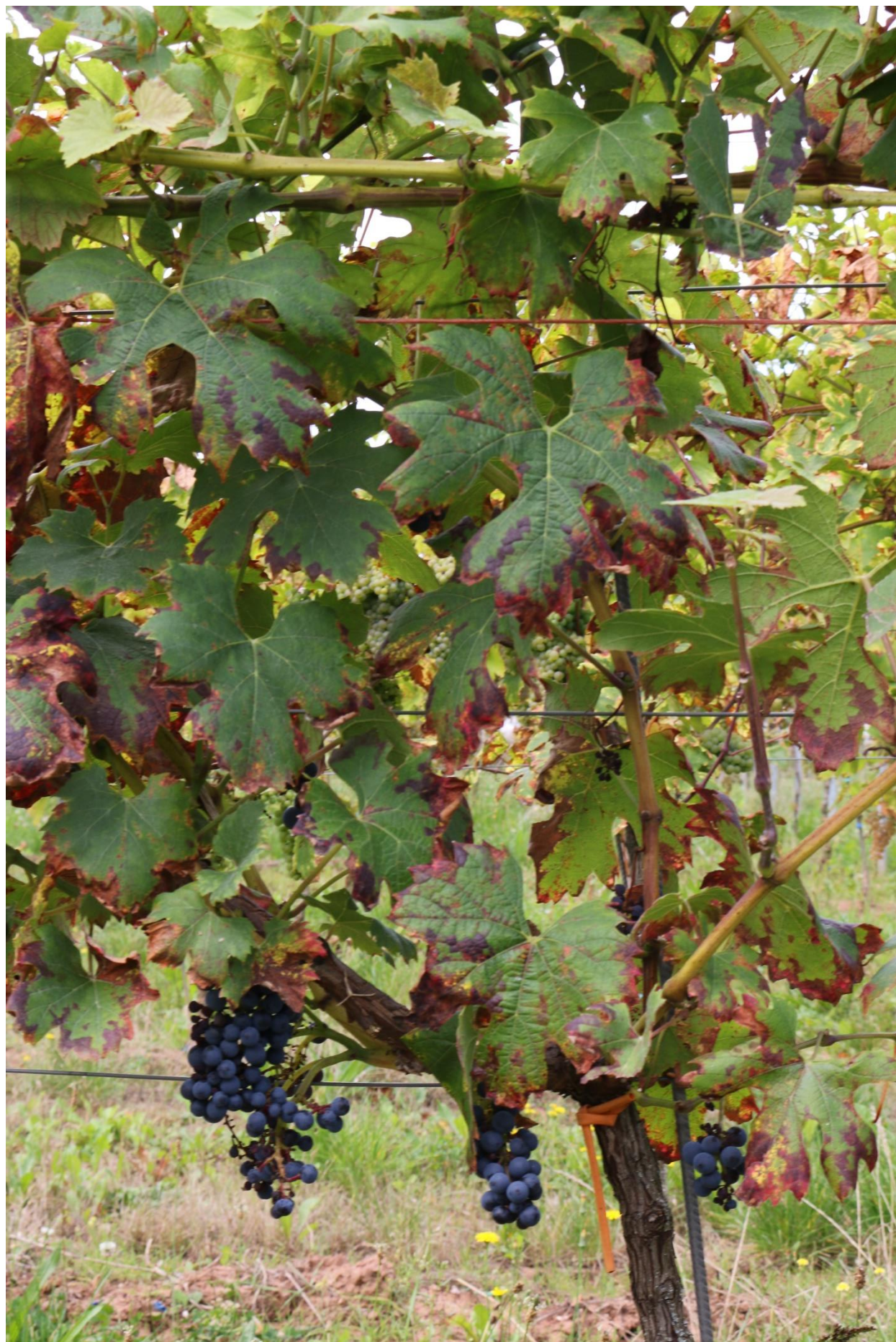

Figure S2: Vine of the LSH line Gf. 2014-087-0015

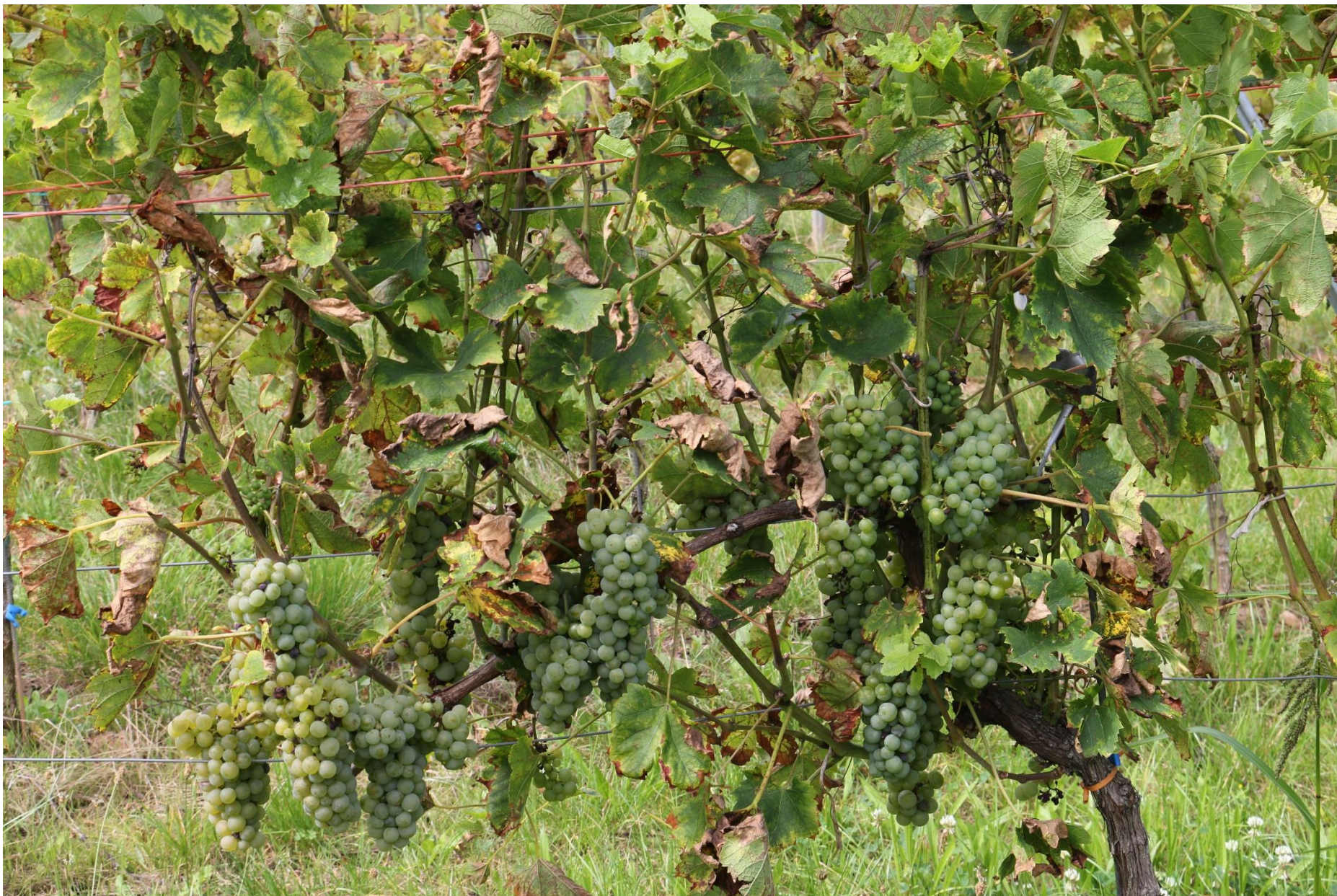

Figure S3: Vine of the LSH line Gf. 2014-092-0051

Supplement: Supplementary file 1 — Supplementary file1 (PDF 1650 kb) [file 122_2022_4077_MOESM1_ESM.pdf]
